# Targeting Neuroinflammation with Abscisic Acid Reduces Pain Sensitivity in Females and Hyperactivity in Males of an ADHD Mice Model

**DOI:** 10.3390/cells12030465

**Published:** 2023-01-31

**Authors:** María Meseguer-Beltrán, Sandra Sánchez-Sarasúa, Marc Landry, Nora Kerekes, Ana María Sánchez-Pérez

**Affiliations:** 1 Insitute of Advanced Materials (INAM), University of Jaume I, 12071 Castellon, Spain; 2 Faculty of Health Sciences, University of Jaume I, 12071 Castellon, Spain; 3 University of Bordeaux, CNRS, Institute for Neurodegenerative Diseases, UMR 5293, F-33000 Bordeaux, France; 4 Department of Health Sciences, University West, 46186 Trollhättan, Sweden

**Keywords:** attention deficit/hyperactivity disorder (ADHD), microglia, oxidative stress, Ape1, pain sensitivity, hyperactivity, novel object recognition, spatial memory, neuroinflammation

## Abstract

Attention deficit/hyperactivity disorder (ADHD) is a neurodevelopmental syndrome characterized by dopaminergic dysfunction. In this study, we aimed to demonstrate that there is a link between dopaminergic deficit and neuroinflammation that underlies ADHD symptoms. We used a validated ADHD mice model involving perinatal 6-OHDA lesions. The animals received abscisic acid (ABA), an anti-inflammatory phytohormone, at a concentration of 20 mg/L (drinking water) for one month. We tested a battery of behavior tests, learning and memory, anxiety, social interactions, and pain thresholds in female and male mice (control and lesioned, with or without ABA treatment). Postmortem, we analyzed microglia morphology and Ape1 expression in specific brain areas related to the descending pain inhibitory pathway. In females, the dopaminergic deficit increased pain sensitivity but not hyperactivity. In contrast, males displayed hyperactivity but showed no increased pain sensitivity. In females, pain sensitivity was associated with inflammatory microglia and lower Ape1 levels in the anterior cingulate cortex (ACC) and posterior insula cortex (IC). In addition, ABA treatment alleviated pain sensitivity concomitant with reduced inflammation and normalized APE1. In males, ABA reduced hyperactivity but had no significant effect on inflammation in these areas. This is the first study proving a sex-dependent association between dopamine dysfunction and inflammation in specific brain areas, hence leading to different behavioral outcomes in a mouse model of ADHD. These findings provide new clues for potential treatments for ADHD.

## 1. Introduction

Attention deficit/hyperactivity disorder (ADHD) is a neurodevelopmental syndrome affecting 5–10% of children and 2.5% of adults worldwide [1]. ADHD often co-occurs with other psychiatric conditions (i.e., autism spectrum disorder, anxiety, and behavioral problems) [2] and somatic complaints (e.g., asthma [3], celiac disease [4], and migraine [5]), which are often also diagnosed alongside ADHD [6]. ADHD deeply affects patients’ functioning and quality of life (QoL) [7,8,9]. ADHD’s social impact is also relevant: throughout an individual’s lifetime, ADHD can increase the risk of other psychiatric disorders, educational and occupational failure, accidents, criminality, social disability, and addictions [1,10]. ADHD is usually diagnosed in boys and girls at a ratio of approximately 2:1, here showing clear phenotypical differences [11]. A recent meta-analysis revealed that girls do not often display symptoms of hyperactivity and impulsivity, which are more often recognized in boys. However, the executive deficits present in ADHD compared with typically developed controls are equally distributed among girls and boys [12].

The heritability of ADHD is high [13], but environmental factors greatly contribute to its onset and development [14]. Growing evidence points to the influence of inflammation [15,16], microbial dysbiosis [17], and maternal autoimmune reactions [18] that can affect ADHD co-existing conditions such as anxiety [19] and possibly pain [20]. Systemic inflammation is increasingly acknowledged as a common denominator for psychiatric disorders [21], but the mechanism by which a general inflammatory situation that causes neuroinflammation leads to specific psychiatric symptoms remains elusive. 

Neuroinflammation can induce brain dysfunction, and it is consistently found in neurological and psychiatric disorders [22,23]. Increased inflammation during early neurodevelopment is suspected to be a risk factor aggravating and/or triggering ADHD symptoms [24], though no mechanism has yet been proposed. Epidemiological studies, including meta-analyses, have revealed that patients with ADHD are more likely to suffer other inflammatory conditions (e.g., asthma, allergic rhinitis, atopic dermatitis, and allergic conjunctivitis) [16,25,26]. Moreover, maternal inflammatory status can prompt the occurrence of ADHD and other neurodevelopmental disorders in offspring [27,28,29].

Attention and pain are intimately related [30,31,32]. In humans, evidence indicates that ADHD increases pain perception in adults [33,34], and the prevalence of generalized pain is higher in ADHD patients (up to 80%) compared with the control population (17%) [35,36]. Neuroanatomical studies have shown that attention and pain transmission use the same neural networks. One of these networks, the anterior cingulate cortex (ACC), which receives sensitive information via the thalamus and projects to the insular cortex (IC), has been well characterized in regulating attention and pain [37,38]. ACC hyperexcitability is associated with higher levels of pain [39], and glia activation can trigger hyperexcitability modulating neuronal channel function [40]. Furthermore, inflammatory markers in ACC are linked to pain sensitization [41,42,43]; thus, we aimed to test whether dopaminergic dysfunction underlying ADHD symptoms could increase ACC inflammation. If this were the case, reducing inflammation would alleviate ADHD symptomatology. To this end, we evaluated microglia morphology and oxidative stress markers in the ACC and IC in relation to behavior in a previously validated mouse model of ADHD [44]. We have formerly shown the beneficial effects of abscisic acid (ABA) as an anti-inflammatory molecule in rodent models of Alzheimer’s disease and metabolic syndrome [45,46]. Therefore, we tested the effects of ABA alleviating ADHD symptomatology. Animal models of ADHD have mostly focused on male subjects, but in the present study, we studied both females and males to discern whether the differences in symptoms in humans have biological grounds or whether they are because of gender-stereotypical expressions of common pathophysiology.

## 2. Materials and Methods

### 2.1. Animals and Surgical Procedures

**Figure 1:**
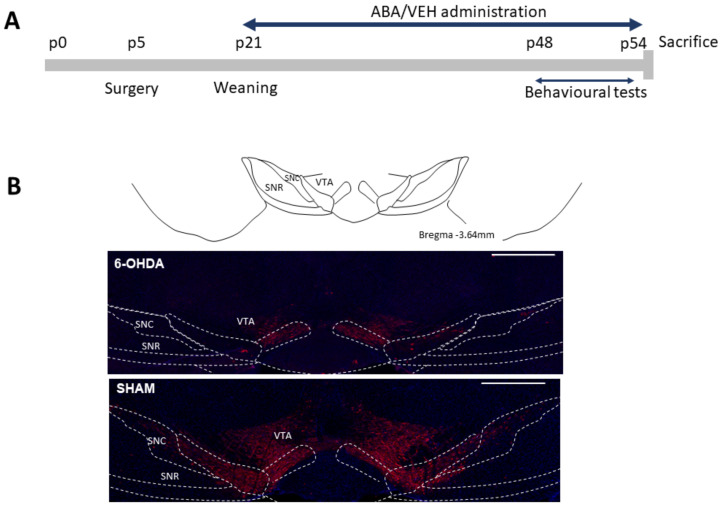
Experimental design. (**A**) Timeline of the experiment. Surgery (inoculation of 6-OHDA or sham into lateral ventricle AP −2 mm, ML ±0.6 mm, DV −1.3 mm from Bregma) was performed on postnatal day 5 (P5). ABA or vehicle administration started at weaning on postnatal day 21 (p21) for one month. Behavioral tests were carried out for one week before terminating the experiment. (**B**) VTA and SN. VTA, ventral tegmental area; SNC, substantia nigra compact part; SNR, substantia nigra, reticular part. Calibration bar: 500 µm.

Female and male Swiss mice (Janvier-Labs; Saint-Berthevin, France) were kept at the animal facility of the University Jaume I. The procedures followed European community norms on the protection of animals used for scientific purposes. The experiments were approved by the Ethics Committee of the University of Jaume I (scientific procedure 2020/VSC/PEA/0099). The animals were maintained on a 12 h:12 h light cycle and provided with food and water ad libitum. The pups were housed with their mothers and kept in constant temperature conditions (24 °C ± 2). After weaning, the animals were housed in groups of two to four mice to reduce isolation-induced stress. Sixty-nine mice were randomly assigned to the sham or 6-OHDA groups. At postnatal day 5 (P5), the pups received 6-OHDA hydrobromide (Merck KGaA, Darmstadt, Germany) or a vehicle in one of the lateral ventricles, as described in [44]. Thirty minutes before surgery, the mice were injected with desipramine hydrochloride pretreatment (20 mg/kg sc; Merck KGaA, Darmstadt, Germany) to inhibit the norepinephrine transporter in noradrendergic neurons. The mice were anesthetized with 3% isoflurane and maintained with 0.8% isoflurane during surgery. After this, 25 μg of 6-OHDA dissolved in 3 μL ascorbic acid 0.1% or vehicle were infused into one of the lateral ventricles (stereotaxic coordinates: AP −2 mm, ML ±0.6 mm, DV −1.3 mm from Bregma) [47]. After weaning (P21), the mice were randomly administered either ABA in their drinking water (20 mg/L) or a vehicle. Animal welfare was monitored throughout the procedure according to ethical committee rules (timeline, Figure 1A).

### 2.2. Behavioral Procedures

In all the procedures, the mice were habituated to the testing room 30 min before performing each behavior paradigm. The animals were recorded using a video tracking system (ANY-maze, Stoelting Europe, Dublin, Ireland). All behavioral procedures were conducted in dim light. Behavioral tests started at P48, one week before completing the ABA treatment. The researcher was blinded to the group’s condition. All apparatuses and objects were cleaned using a 30% ethanol solution between subjects.

Spontaneous locomotor activity was assessed in the open field arena (Ugo Basile, Germonio (VA), Italy). The mice were placed in an open field facing one of the walls and allowed to freely explore for 10 min. Distance traveled (cm) and speed (cm/s) were quantified. 

Recognition memory was assessed using a novel object recognition (NOR) test. The NOR experiment was conducted as described previously [46]. On the test day, the subjects were left to explore two identical objects for 10 min (familiarization phase). After 30 min, the mice were sent back to the arena and allowed to explore one of the previous objects (familiar) and a novel object for 10 min (test phase). In the present study, “exploration” has been defined as sniffing or touching the object (head oriented toward it). Data were expressed as the discrimination index (DI) ((Time spent on the novel object―Time spent on the familiar object)/Total exploration time)). 

Spontaneous alternation was measured using the T-maze test, as described previously [48]. Briefly, the animal was placed in the starting arm and left to explore for 5 min, with access to two of the three arms (familiarization phase). The mice were then returned to the home cage for a 30 min intertrial interval and then placed back in the starting position but now with free access to all three arms for 5 min (test phase). The arm that was closed during the familiarization phase was considered the “novel” arm, and the arm visited during the familiarization phase was considered the “familiar” arm. Data were expressed as DI ((Time spent on the novel arm − Time spent on the familiar arm)/Total exploration time)). 

Anxiety was measured through an elevated plus maze *(EPM*) (Ugo Basile, Germonio (VA), Italy). The EPM is an elevated nonreflective metal with two closed arms (35 × 15 × 60 cm). As described previously [49], all mice were placed in the center of the maze and allowed to run freely around the maze for 10 min. Data were expressed as the percentage of time spent exploring the open arms compared with the total time in the EPM. 

Social interaction was evaluated in a three-chamber social interaction paradigm. The animals were placed in the central chamber and allowed to freely explore all chambers for 10 min (habituation phase). Immediately after the habituation phase, we performed the test phase (10 min). The mouse was allowed to explore either an object or a con-specific mice protected by a fence placed in the lateral chambers. The chamber in which the con-specific object was placed was balanced. In addition, we used four different con-specifics (two males and two females) to balance them. Data were expressed as the percentage of time spent exploring the con-specific mice compared with the total time spent exploring. Climbing or running around it was not considered exploration. 

Pain sensitivity by mechanical stimulus (Von Frey test). The mechanical nociceptive response was assessed using the Von Frey test (Ugo Basile, Germonio (VA), Italy), as described previously [50]. Briefly, the mice were placed in test individual cages with a mesh floor for 30 min (habituation). The withdrawal threshold of the paw was set by calibrated Von Frey filaments applied to the ventral surface of the hind paws. Three to five measurements (each paw) were registered, with an interval of 30 s between each. The pain threshold is the grams of the filament at which the mouse withdrew its paw.

Pain sensitivity by thermal stimulus (Plantar test). The thermal nociceptive response was assessed using the Plantar test (Ugo Basile, Germonio (VA), Italy). The mice were placed in testing cages with a glass pane floor for 30 min (habituation). Using an infrared (IRed) generator, the ventral surface of the left and right hind paws was stimulated with an IRed intensity of 50. The cut-off time for finishing the IRed stimulation was set at 15 s. The latency to paw withdrawal was recorded, and five measurements per paw were performed with an interval of 2 min. 

### 2.3. Immunofluorescence Procedure

Immunofluorescence was performed as previously described [51]. Briefly, the mice were anesthetized and perfused with saline (0.9% NaCl), followed by a fixative (4% paraformaldehyde in 0.1 M PB, pH 7.4). After perfusion, the brains were removed, postfixed overnight, and cryoprotected in 30% sucrose in 0.01 M PBS pH 7.4 for 3 days. The brains were cut in the rostro caudal direction (40 μm) using a sliding microtome, Leica SM2010R (Leica Microsystems, Heidelberg, Germany). The primary antibodies mouse anti-Tyrosine Hydroxylase (Merck KGaA, Darmstadt, Germany; 1:5000), rabbit anti- Iba1 (FUJIFILM Wako Chemicals Europe GmbH, Neuss, Germany 1:1000), and mouse anti-Ref1/Ape1 (Santa Cruz Biotechnology, Santa Cruz, CA, USA; 1:200) were incubated overnight. Next, the sections were rinsed and incubated for 2 h at RT with donkey antimouse Cy3 or donkey antirabbit Alexa 488 secondary antibodies (Jackson Immunoresearch, Suffolk, UK). Finally, the sections were mounted on slides and covered using Fluoromount-G mounting medium (Invitrogen, Waltham, MA, USA).

Imaging and analysis. Fluorescence images were taken with a confocal scan unit with a module TCS SP8 equipped with argon and helio-neon laser beams attached to a Leica DMi8 inverted microscope (Leica Microsystems). Excitation and emission wavelengths for Cy3 were 433 and 560–618 nm, respectively; Alexa488 labeled excitation wavelength was 488 nm, and its emission was 510–570 nm. For quantification of Tyrosine Hydroxylase labeling, we used a 10× lens. Image J software was used to count Tyrosine Hydroxylase labeling. Data were expressed as the percentage of Tyrosine Hydroxylase labeling with respect to the sham group. For the quantification of Iba1 labeling, we used 20× lens. Image J software combined with a FracLac plugin [52] was used to analyze the microglia morphology in sections from the sham and 6-OHDA groups of both females and males, as previously described [46]. The microglia morphological parameters that were analyzed were (1) cell area, meaning the total number of pixels corresponding to the area occupied by the cell, soma, and branches, and (2) cell perimeter, which was based on the single outline cell shape. For the quantification of Ref1/Ape1 labeling, we used a 20× lens. Data were expressed as the mean of gray values per area (insula and ACC). 

### 2.4. Statistical Analysis

The analysis was carried out with Graph Pad software (GraphPad Prism V8 software, GraphPad, La Jolla, CA, USA). Data were subjected to the Shapiro–Wilk test for Gaussian distribution. If normality was confirmed, data were reported as the mean ± SEM and the “n” the number of independent subjects. For differences in either, a two-way ANOVA was carried out to evaluate the interaction between lesion and ABA treatment, which was followed by a Tukey’s multiple comparison test. A two-tailed unpaired Student’s *t*-test with the probability set at α < 0.05 was used in specific cases where one variable (either lesion or ABA) was compared in two groups. This is indicated by a § symbol in the graphs.

## 3. Results

### 3.1. 6-OHDA Reduced TH Staining in the Ventral Tegmental Area

To ascertain the level of dopaminergic lesions induced by 6-OHDA injection, we carried out TH immunodetection (Figure 1B). We confirmed that, in the sham surgery animals, TH expression was detected in the ventral tegmental area (VTA) and adjacent substantia nigra (SN). Animals with less than 45% lesions were discarded from the analysis.

The percentage of TH reduction below the cut-off was similar for females and males (Figure S1). 

### 3.2. Effect of Perinatal i.c.v. 6-OHDA Injection and ABA Treatment on Spontaneous Locomotor Activity

**Figure 2:**
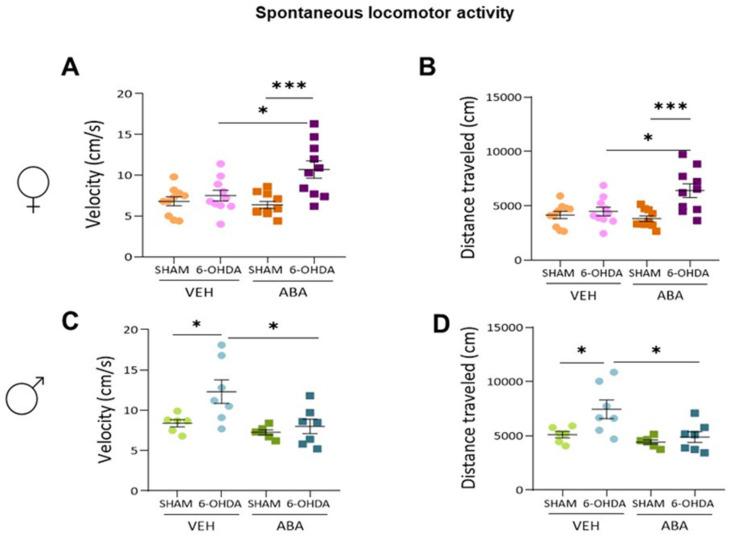
Spontaneous locomotor activity. (**A**) Speed (cm/s) and (**B**) distance (cm) in females. (**C**) Speed (cm/s) and (**D**) distance (cm) in males. Data presented as mean ± SEM (*n* = 6–11 per condition) and analyzed by two-way ANOVA followed by Tukey’s multiple comparison test (* *p* < 0.05; *** *p* < 0.001.

The spontaneous locomotor activity in females was measured by velocity (Figure 2A) and distance traveled (Figure 2B). Two-way ANOVA analysis revealed that there was an interaction between lesion and ABA treatment (F _(1,36)_ = 6.42, *p* = 0.0158, and F _(1,36)_ = 6.86, *p* = 0.0128, respectively). Tukey’s multiple comparison test revealed that the lesions affected velocity only in ABA-treated mice (6-OHDA-ABA vs. SHAM-ABA: *q* = 6.057, *p* = 0.0007, ***) and that ABA treatment increased velocity only in lesioned females (6-OHDA-VEH vs. 6-OHDA-ABA: *q* = 4.456, *p* = 0.0165, *). Similarly, distance traveled (Figure 2B) was affected by the lesion only in ABA-treated mice (6-OHDA-ABA vs. SHAM-ABA: *q* = 6.015, *p* = 0.0008, ***), and ABA increased the distance traveled only in lesioned mice (6-OHDA-VEH vs. 6-OHDA-ABA: *q* = 4.475, *p* = 0.0159, *). 

On the contrary, in male velocity (Figure 2C), no interaction between lesion and ABA was found, but a strong effect of lesion (F_(1,22)_ = 5.813, *p* = 0.0247) and ABA (F_(1,22)_ = 7.986, *p* = 0.0098) was shown. Similarly, distance traveled (Figure 2D) was increased by lesion (F_(1,22)_ = 6.129, *p* = 0.0215), and ABA treatment reduced it (F_(1,22)_ = 8.378, *p* = 0.0084). Tukey’s multiple comparison test confirmed that lesions increased spontaneous activity in the untreated group, velocity (6-OHDA-VEH vs. SHAM-VEH: *q* = 4.061, *p* = 0.041, *) and distance traveled (6-OHDA-VEH vs. SHAM-VEH: *q* = 4.171, *p* = 0.0347, *). Importantly, ABA treatment prevented the lesion-induced increase both in velocity (6-OHDA-ABA vs. 6-OHDA-VEH: *q* = 4.659, *p* = 0.0162) and distance traveled (6-OHDA-VEH vs. 6-OHDA-ABA: *q* = 4.778, *p* = 0.0134, *).

### 3.3. 6-OHDA Injection Reduces Novel Object Recognition, and ABA Treatment Does Not Rescue the Lesion Effect

**Figure 3:**
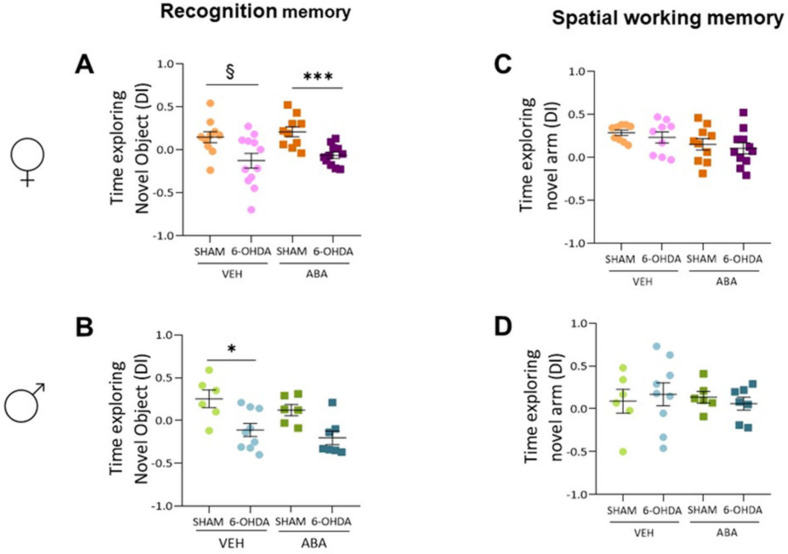
Recognition and spatial working memory. (**A**) Time exploring (d-index) the novel object in the novel object recognition test in females and (**B**) males. (**C**) Time (d-index) exploring the novel arm in the T-maze test in females and (**D**) males. Data are expressed as a discrimination index ((Time exploring novel – time exploring familiar)/total time exploring), presented as mean ± SEM (*n* = 6–11 per condition) and analyzed using a two-way ANOVA and Tukey’s test (* *p* < 0.05, *** *p* < 0.001). § *p* < 0.05 the difference within the same treatment is analyzed by Student’s *t*-test.

In females (Figure 3A), lesions affected the NOR (F_(1,37)_ = 14.94, *p* = 0.0004), but ABA had no effect; in addition, there was no interaction between lesions and treatment. Tukey’s multiple comparison test identified significant lesion-induced differences only in ABA-treated mice (6-OHDA-ABA vs. SHAM-ABA: *q* = 4.055, *p* = 0.0329, *). To evaluate the effect of lesions only in untreated females, we applied an unpaired Student *t*-test, which revealed that the lesions reduced the discrimination index (*p* = 0.023, t = 2.465 df = 20). 

In males (Figure 3B), the two-way ANOVA revealed that lesions had a strong overall effect (F_(1,24)_ = 16.98, *p* = 0.0004), whereas for ABA, lesions did not have a strong effect. No interaction between lesions and ABA was detected. Tukey’s multiple comparison test indicated that lesions had an effect only in untreated mice (6-OHDA-VEH vs. SHAM-VEH: *q* = 4.483, *p* = 0.0201, *).

### 3.4. The 6-OHDA Lesion Did Not Alter Spatial Memory as Measured by Spontaneous Alternation (T-Maze) Nor Anxiety as Measured by Elevated plus Maze

In females (Figure 3C), the two-way ANOVA analysis showed that lesions did not affect spontaneous alternation, and surprisingly, ABA treatment had a significant effect (F_(1,35)_ = 4.814, *p* = 0.0350). There was no interaction between lesions and ABA treatment, and Tukey’s multiple comparison test did not show specific differences. On the other hand, in males (Figure 3D), a two-way ANOVA confirmed that lesions and ABA treatment had no effect on spontaneous alternation, suggesting a sex-dependent effect of ABA.

### 3.5. The 6-OHDA Lesion Had a Sex-Dependent Effect on Pain Sensitivity Tests—ABA Treatment Had a Potential Beneficial Effect Elevating Pain Sensitivity Threshold

**Figure 4:**
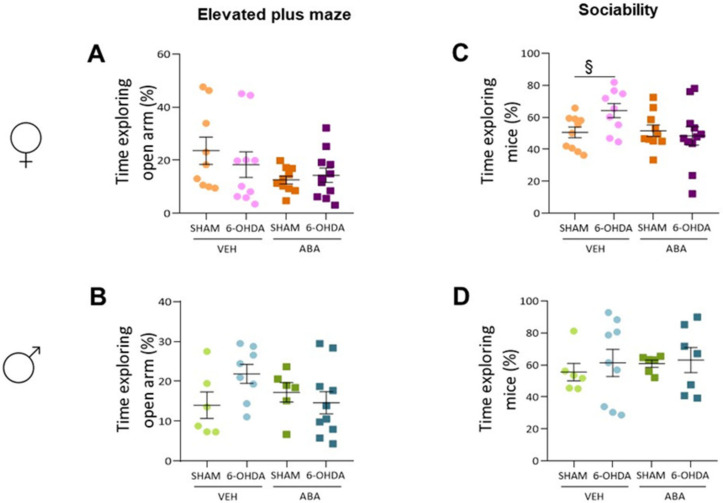
Anxiety and sociability. (**A**) Percentage of time exploring the open arms in the elevated plus maze test in females and (**B**) males. (**C**) Percentage of time exploring the con-specific mice in the three-chamber test in females and (**D**) males. Data presented as mean ± SEM (*n* = 6–11 per condition) and analyzed using two-way ANOVA and Tukey’s test. § indicates significant difference (§ *p* < 0.05) within the same treatment, by Student’s *t*-test.

The time exploring open arms was not affected in females (Figure 4A) by lesion or treatment. Similarly, lesion or treatment did not affect the EPM in males (Figure 4B). 

### 3.6. Perinatal Injection of 6-OHDA Lesions Had a Sex-Dependent Effect on Sociability in Two-Month-Old Mice

Females’ sociability (Figure 4C) was analyzed by two-way ANOVA, which indicated no effect of 6-OHDA lesion or ABA treatment. However, comparing the effect of lesions only, the Student’s *t*-test revealed that lesion increased time exploring the con-specific (*p* = 0.023, t = 2.500, df = 17); this difference was not maintained in ABA-treated mice, suggesting that ABA may reverse the lesion effect. In males (Figure 4D), the two-way ANOVA indicated no effect of lesion or ABA treatment. Student’s *t*-test did not show significant differences. 

### 3.7. Perinatal 6-OHDA Lesion Had a Sex-Dependent Effect on Pain Sensitivity by the Mechanical Stimulus, as Measured by the Von Frey Test

**Figure 5:**
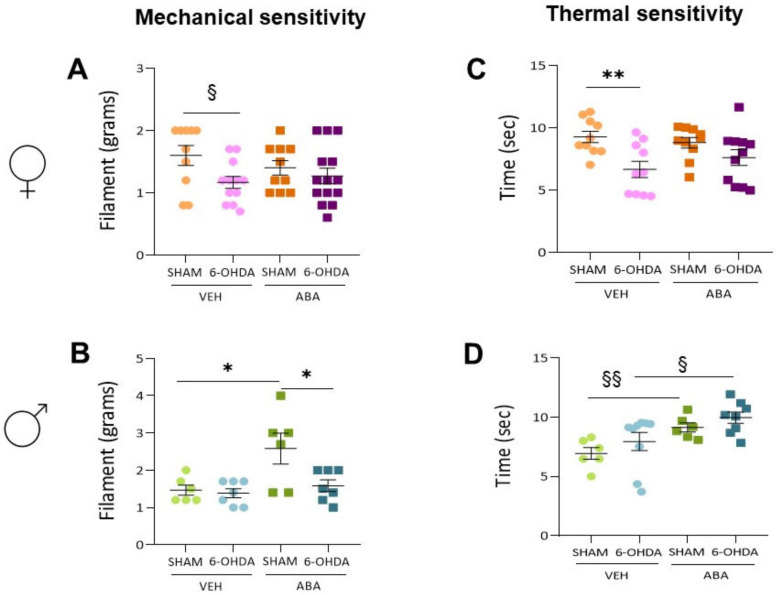
Thermal and mechanical sensitivity. (**A**) Threshold (grams) after application of mechanical stimulus in the Von Frey test in females and (**B**) males. (**C**) Threshold (s) after application of thermal stimulus in the Plantar Test in females and (**D**) males. Data presented as mean ± SEM (*n* = 6–11 per condition) and analyzed using two-way ANOVA and post hoc multiple comparison Tukey’s test (* *p* < 0.05, ** *p* < 0.01). § indicates significant differences (§ *p* < 0.05; §§ *p* <0.01) within the same treatment, analyzed by Student’s *t*-test.

In females (Figure 5A), the two-way ANOVA indicated that 6-OHDA lesions increased mechanical sensibility (F_(1,42)_ = 4.974, *p* = 0.0311), although ABA did not have an overall effect and no interaction was found. Tukey’s multiple comparison tests showed no specific differences. However, to understand the effect of lesions only, the Student’s *t*-test revealed that lesions induced a significant reduction in pain threshold only between vehicle groups (*p* = 0.025, t = 2.424, df = 20), not in the ABA-treated groups, suggesting that ABA could counteract the 6-OHDA lesion effect.

In males (Figure 5B), the two-way ANOVA showed that lesion had no effect, but ABA did (F_(1,22)_ = 8.327, *p* = 0.086), and there was an interaction between them (F_(1,22)_ = 4.036, *p* = 0.0570). Tukey’s multiple comparison test revealed that ABA increased the pain threshold only in sham mice (SHAM-VEH vs. SHAM-ABA: *q* = 4.717, *p* = 0.0147), whereas in lesion mice, the threshold with the ABA threshold was low again (6-OHDA-ABA vs. SHAM-ABA: *q* = 4.373, *p* = 0.0254, *), suggesting that lesions with 6-OHDA can prevent the ABA effect.

### 3.8. Perinatal 6-OHDA Lesions Had a Sex-Dependent Effect on Pain Sensitivity by Thermal Stimulus, and ABA Treatment Increased the Threshold in Both Females and Males

In females (Figure 5C), a two-way ANOVA revealed that lesions decreased the pain threshold (F_(1,37)_ = 12.29, *p* = 0.0012); however, ABA treatment did not have an overall effect. The test showed no interaction between lesions and ABA. A post hoc Tukey’s multiple comparison test showed that lesions reduced pain only in untreated females (6-OHDA-VEH vs. SHAM-VEH: *q* = 4.775, *p* = 0.0090, **), suggesting that ABA treatment prevented the lesion effect.

On the contrary, in males (Figure 5D), a two-way ANOVA showed that lesions had no effect, but ABA had an overall effect (F_(1,25)_ = 11.43, *p* = 0.0024). No interaction between these two factors was observed. The post hoc test did not identify individual differences. To understand the effect of ABA, the Student’s *t*-test revealed that ABA improved the sensitivity in the sham (*p* = 0.0058, t = 3.495, df = 10) and 6-OHDA lesions (*p* = 0.0473, t = 2.16, df = 15).

### 3.9. In the Anterior Cingulate Cortex (ACC), the 6-OHDA Lesion Induced Microglia Polarization to a Proinflammatory Status, and ABA Rescued the Effect in Females but Not in Males

**Figure 6:**
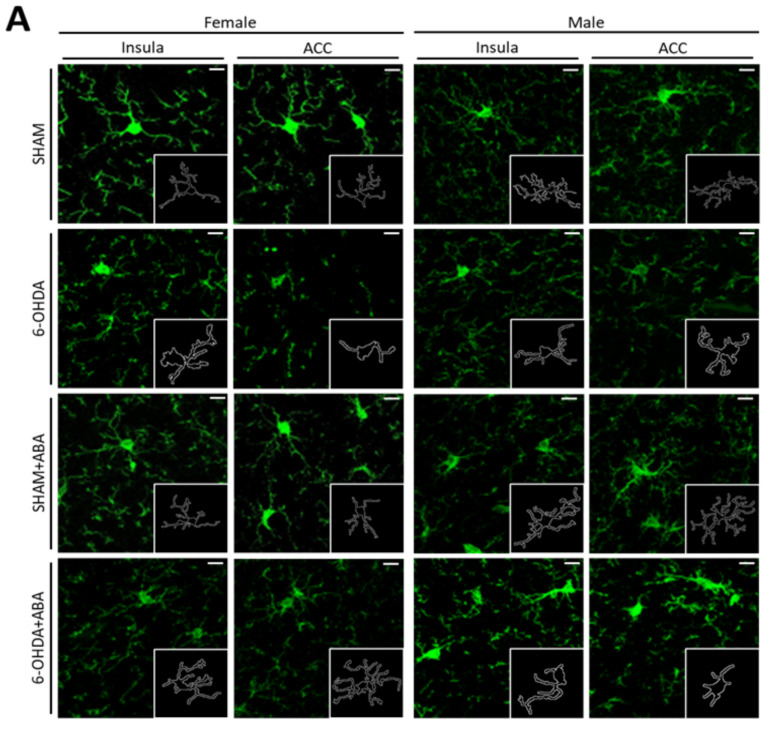

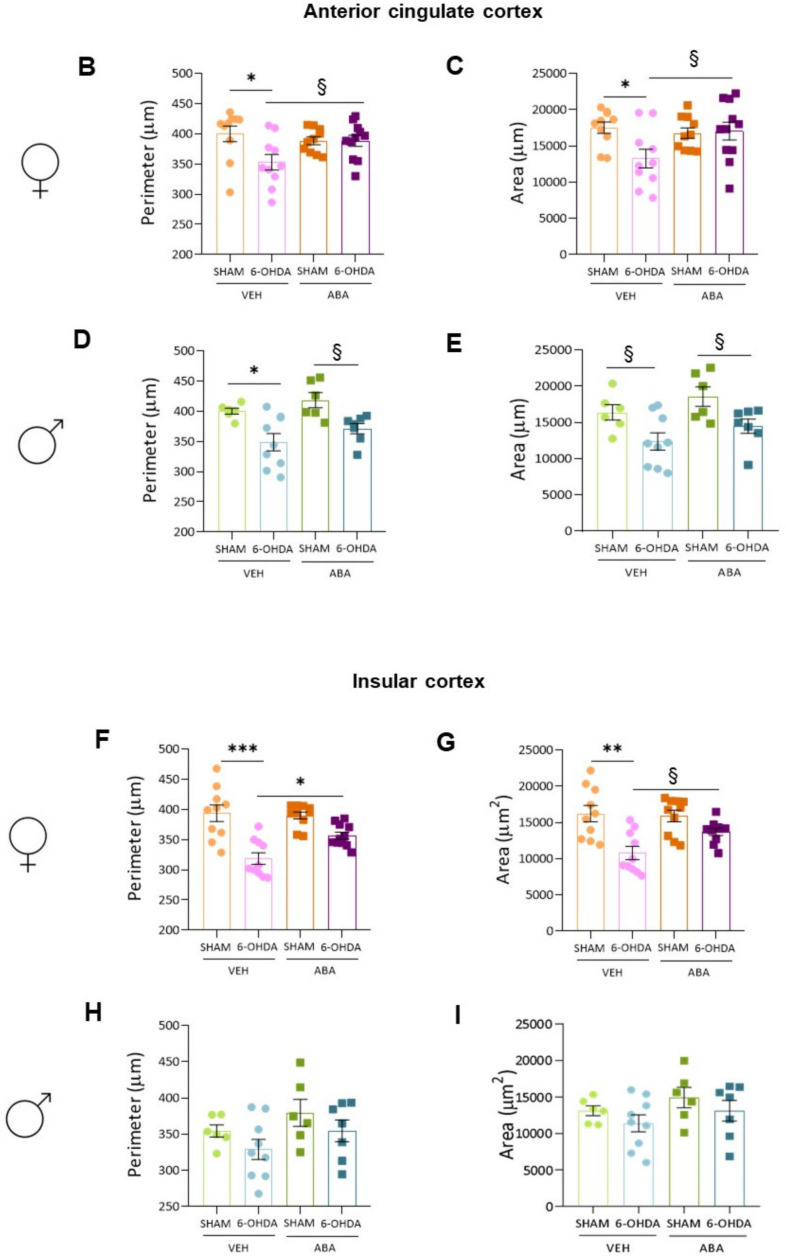
Microglia morphology in the anterior cingulate cortex (ACC) and posterior insular cortex **(pIC).** (**A**) Representative image of microglia in ACC and pIC. (**B**) Perimeter (µm) and (**C**) area (µm^2^) in ACC in females. (**D**) Perimeter (µm) and (**E**) area (µm^2^) in ACC in males. (**F**) Perimeter (µm), (**G**) area (µm^2^) in females’ pIC; (**H**) perimeter (µm) (**I**) area (µm^2^) in males’ pIC. Data are expressed as mean ± SEM (*n* = 6–11 per condition) and analyzed using two-way ANOVA and Tukey’s test (* *p* < 0.05, ** *p* < 0.01,*** *p* < 0.001). § indicates significant differences (§ *p* < 0.05) within lesion, analyzed by Student’s *t*-test. Calibration bar; 10 µm.

Microglia were visualized using Iba-1 staining in the ACC of females and males (Figure 6A). The quantification of microglia morphology (perimeter and area) was carried out. The perimeters in female microglia analyzed by two-way ANOVA indicated a lesion-reduced perimeter (Figure 6B; F_(1,37)_ = 4.808, *p* = 0.0347) and area (Figure 6C; F_(1,37)_ = 7.333, *p* = 0.0102). This indicates a reduction in ramified microglia (M0). No overall effect of ABA was found, and no interaction between factors was found. Tukey’s multiple comparison test revealed that the lesion effect occurred only in the untreated females’ microglia perimeter (Figure 6B (6-OHDA-VEH vs. SHAM-VEH: q = 4.335, *p* = 0.0202, *)) and microglia area (Figure 6C (6-OHDA-VEH vs. SHAM-VEH: *q* = 4.458, *p* = 0.0162, *)), suggesting that ABA prevented a lesion proinflammatory effect. To better understand the effect of ABA only, a two-tailed Student’s *t*-test revealed that ABA significantly improved the microglia perimeter (*p* = 0.0343, t = 2.281, df = 19) and microglia area ((*p* = 0.0462, t = 2.133, df = 19) in lesioned subjects. 

In males’ ACC, the two-way ANOVA revealed that a lesion reduces the perimeter (Figure 6D; F_(1,24)_ = 17.05, *p* = 0.0004) and area (Figure 6E; F_(1,24)_ = 11.49, *p* = 0.0024). ABA had no overall effect, and there was no interaction among the factors in any parameter, perimeter, or area. A Tukey’s multiple comparison picked the significant effect of lesion in the perimeter (6-OHDA-VEH vs. SHAM-VEH: *q* = 4.4268, *p* = 0.022, *). Similarly, in the microglia area, the Student’s *t*-test showed that lesions significantly reduced the area in VEH (*p* = 0.0357, t = 2.825, df = 13). ABA had no effect. This result suggests that, contrary to females, ABA could not counteract the proinflammatory microglia status induced by the 6-OHDA lesion in male’s ACC.

### 3.10. In the Posterior Insular Cortex, 6-OHDA Lesions Induced Microglia Polarization to a Proinflammatory Status Only in Females, and ABA Rescued the Effect

In females’ posterior insula, two-way ANOVA showed a strong 6-OHDA lesion effect in the microglia perimeter (Figure 6F) (F _(1,37)_ = 35.21, *p* < 0.0001). Lesion and ABA treatment had a significant interaction (F_(1,37)_ = 5.216, *p* = 0.0282). Tukey’s multiple comparison test revealed that lesions affected microglia morphology in untreated females (6-OHDA-VEH vs. SHAM-VEH: *q* = 8.124, *p* < 0.0001, ****). Moreover, ABA rescued microglia morphology in 6-OHDA (6-OHDA-VEH vs. 6-OHDA-ABA: *q* = 4.204, *p* = 0.0255, *). Similarly, for the area parameter (Figure 6G), the two-way ANOVA revealed a strong lesion effect (F_(1,36)_ = 19.83, *p* < 0.0001). Tukey’s multiple comparison test confirmed that lesions affected only untreated mice (6-OHDA-VEH vs. SHAM-VEH: *q* = 6.374, *p* = 0.0004, ***). To understand if ABA could rescue the lesion effect, we compared lesions with lesions plus ABA using Student’s *t*-test, which revealed significant differences (*p* = 0.015, t = 2.856, df = 18). On the contrary, in males, we found no significant effect of lesion or ABA treatment in the perimeter (Figure 6H) or area (Figure 6I) when using the two-way ANOVA.

Altogether, these results indicate that in females, but not in males, lesions can induce inflammation in brain areas (ACC and posterior IC). ABA rescues the lesion effect in females and has no effect in males.

### 3.11. Perinatal 6-OHDA Lesion and ABA Treatment Affected APE1 Intensity Differently in the ACC of Females and Males

**Figure 7:**
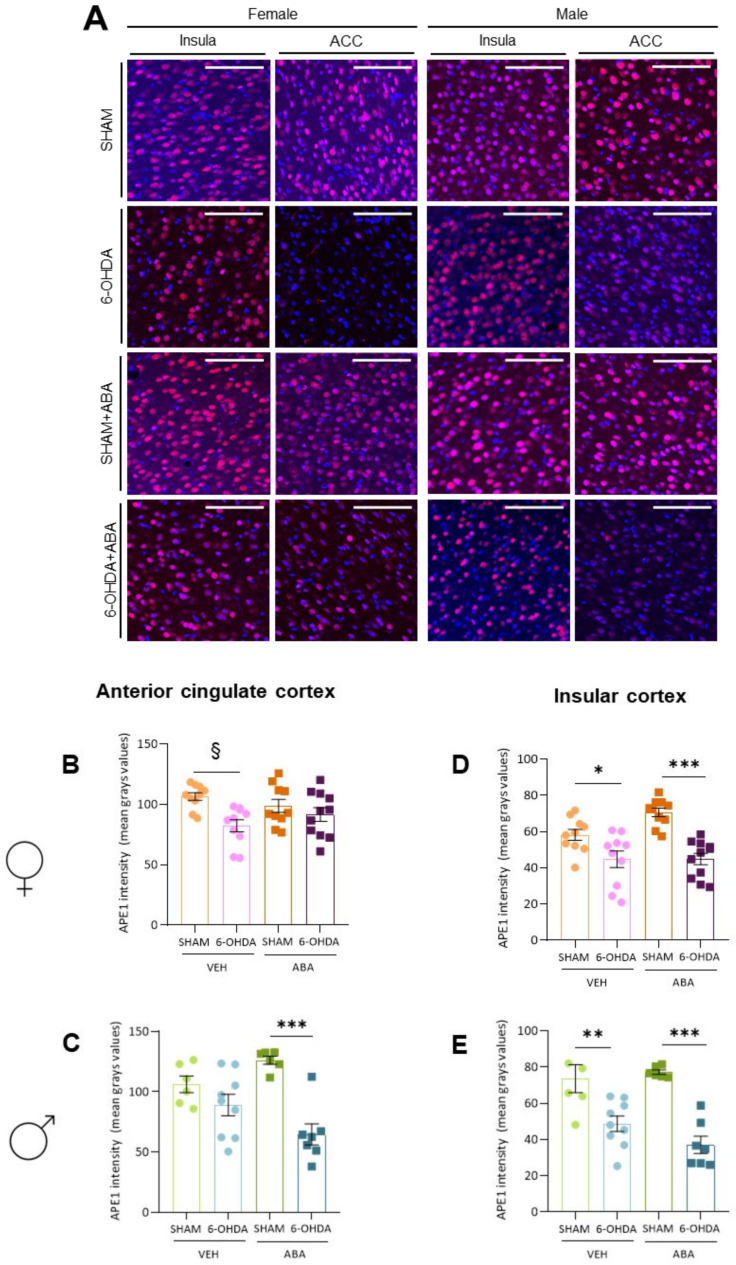
Oxidative stress as measured by APE1/REF1 expression. (**A**) Representative image of APE1/REF1 in the ACC and posterior insula cortex in females and males. (**B**) APE1 intensity in the ACC of females and (**C**) males. (**D**) APE1 intensity in the insula cortex of females and (**E**) males. Data are expressed as mean ± SEM (*n* = 6–11 per condition) and analyzed using a two-way ANOVA and post hoc Tukey’s test, (* *p* < 0.05; ** *p* < 0.01; *** *p* < 0.001). § indicates significant differences (§, *p* < 0.05) within same treatment, analyzed by Student’s *t*-test. Scale bar 100 µm.

Oxidative stress was evaluated by Ape1 immunostaining in the ACC and posterior IC of both female and male mice (Figure 7). Two-way ANOVA analysis showed that, in the ACC of females (Figure 7B), the lesions significantly affected Ape1 intensity (F_(1,36)_ = 6.240, *p* = 0.0172), ABA had no overall effect, and there was no interaction between lesion and ABA treatment. Tukey’s test did not reveal specific further differences. However, to understand the lesion effect, the Student’s *t*-test analysis showed that lesions significantly reduced Ape1 in VEH (*p* = 0.009, t = 3.313, df = 18) but not in ABA-treated female mice (*p* = 0.46, t = 0.748, df = 19), suggesting a potential benefit of ABA administration in females. 

In males (Figure 7C), two-way ANOVA revealed an overall lesion effect (F_(1,26)_ = 24.55 *p* < 0.0001) and no overall ABA effect, but an interaction between the lesion and ABA was found (F_(1,26)_ = 8.5 *p* = 0.0072). Tukey’s test revealed that lesions had a significant effect only in ABA-treated mice (6-OHDA-ABA vs. SHAM-ABA *q* = 9.795, *p* < 0.0001, ****). The interactions between factors were sometimes difficult to interpret, but this may suggest a beneficial effect of ABA in lesioned but not in controls.

### 3.12. Perinatal 6-OHDA Lesion Reduces Ape1 Intensity in Both Females’ and Males’ Posterior Insula

In females (Figure 7D), a two-way ANOVA analysis revealed a strong effect of lesion (F_(1,24)_ = 42.62, *p* < 0.0001) but no overall effect of ABA or interaction. Tukey’s multiple comparisons indicated a significant effect of lesion in untreated subjects (6-OHDA-VEH vs. SHAM-VEH], *q* = 4.1 *p* = 0.029) and treated subjects (6-OHDA-ABA vs. SHAM-ABA *q* = 8.009, *p* < 0.0001, ****), indicating that it cannot rescue the lesion effect. Curiously, ABA increased APE1 intensity only in the sham (VEH-SHAM vs. ABA-SHAM, *q* = 6.735, *p* = 0.0002 ***) but showed no effect in lesioned females.

In males’ insula (Figure 7E), two-way ANOVA showed a strong lesion effect (F_(1,24)_ = 42.62, *p* < 0.0001) but no overall ABA effect or interaction. Tukey’s multiple comparisons analysis revealed that lesions had a significant effect on VEH (6-OHDA-VEH vs. SHAM-VEH, *q* = 5.1 *p* = 0.0067) and ABA (6-OHDA-ABA vs. SHAM-ABA *q* = 7.846, *p* < 0.0001, ****). 

## 4. Discussion

ADHD has been associated with dopamine (DA) system dysfunction for a long time [53,54]. Dopaminergic alterations can increase inflammatory conditions [55]. DA synthesis and metabolism occur not only in neurons but also in microglia, astrocytes, and oligodendrocytes. Moreover, these non-neuronal cells express functional D1 and D2 receptors (for a review, see [56]). Not surprisingly, ADHD has been associated with inflammatory disorders [16,24], and patients with ADHD often suffer from comorbidities of inflammatory etiology [25,26]. 

In our study, we tested the hypothesis that behavioral alterations (hyperactivity, defects in memory, social interaction, and pain sensitivity [20]) resulting from dopaminergic depletion in the VTA, induced by perinatal i.c.v. injection of 6-OHDA [44], are associated with neuroinflammation and oxidative stress in specific brain areas. Furthermore, we hypothesized that targeting neuroinflammation would ameliorate the symptoms.

To target neuroinflammation, we administered ABA to mice in the drinking water for one month. We have previously published the beneficial effects of ABA as a safe anti-inflammatory treatment in rodent models of neurological alterations associated with metabolic syndrome [45,57] and Alzheimer’s disease mutations [46]. 

In the present study, we found a sex-dependent effect of the dopaminergic lesion and ABA administration. We found that dopaminergic lesion increased spontaneous activity in two-month-old males, which is in agreement with the findings in adolescent male mice [44,58] and in the DAT-KO rats (a model of hyperdopaminergia) [59]. Interestingly, the same lesion did not affect the locomotor activity of females. This result may agree with human behavior, where hyperactivity is found to be more prevalent in boys than girls [60]. One month of ABA treatment reduced spontaneous locomotor activity in males, suggesting a potential beneficial effect in managing hyperactivity. This result aligns with the effect that curcumin, a well-known anti-inflammatory molecule, exerts an effect on a hypertensive rat model (considered a spontaneous ADHD model), also reducing hyperactivity in males [61]. However, we observed that ABA increased spontaneous activity in the lesioned females, suggesting an interaction between the lesion and ABA. To the best of our knowledge, this is the first comparative study to suggest a sex-dependent effect of the dopaminergic lesion and the response to an anti-inflammatory intervention on an ADHD model. Further comparative studies are warranted to understand these sex-dependent differences, hence urging a revision of treatment in humans’ patients and further supporting the need for precision medicine. 

We observed that perinatal 6-OHDA injection reduced the NOR in two-month-old females and males, confirming previous reports in one-month-old male mice [44]. The NOR test evaluates recognition memory in mice as a marker of healthy development. However, one month of ABA treatment did not rescue this impairment. We hypothesized that longer treatments may be required, as we previously observed [46]. 

Furthermore, in our model, the dopaminergic lesion, although affecting the NOR, had no effect on spontaneous delayed alternation, which is a measure of spatial working memory that brings together exploratory behavior and cognitive function. Previous reports have validated impaired executive function in the 6-OHDA-induced lesion model through the use of other tests, such as latent inhibition and lower attention in five-choice serial reaction [58]. Our result, however, could not strengthen the role of dopaminergic lesions on spatial working memory. This could be explained by several factors because exploratory behavior and cognitive function depend on a variety of brain regions [62] that are not directly affected by the dopaminergic lesion. 

We observed no differences in the anxiety levels of 6-OHDA injected mice compared with the sham controls, here as measured by 10 min sessions on the EPM. This result contrasts those of previous studies, where differences in anxiety levels were found in lesioned males [44]. To rule out the different times used in other studies, we also measured the EPM behavior in the first 5 min of the EPM session (Figure S2), producing a similar result. We hypothesize that the different results could be attributed to the age differences of the mice used in both studies. In [44], one-month-old mice were used, whereas we used two-month-old mice. Furthermore, anxiety is highly dependent on the environment; in fact, anxiety has been found to co-occur with ADHD core symptoms in approximately 50% of all cases [63]. Thus, we cannot rule out external factors, even those related to animal housing, to explain the observed differences between the studies.

In sociability tests, we observed that 6-OHDA lesioned females interacted more with con-specific than controls, whereas lesions did not affect males, suggesting an interaction between dopaminergic alterations, social functioning, and sex. This feature has not been previously described because no female studies have been carried out in rodent models of ADHD. In humans, deficits in sociability have been associated with ADHD in both genders [64,65], whereas other studies have reported no differences [66]. Further studies are warranted to test social interactions in female and male animal models to understand whether social deficits are a direct consequence of dopaminergic dysfunction or indirectly so as a result of self-perceived stigma in humans.

We found that perinatal lesions increased pain sensitivity to thermal and mechanical stimulus in two-month-old female but not male mice. Although sensory over-responsiveness [67,68] and emotional alterations [69,70] are often found in ADHD children, altered pain sensitivity has been reported mostly in women [33]. Other clinical studies have shown evidence of an association between higher pain sensitivity and ADHD in boys [71] and adults with ADHD [35,36,72]. However, other studies have reported the opposite [73]. 

In addition, preclinical studies have shown different results in male mice; for example, the male rat model reported no pain sensitivity to mechanical stimuli but reported pain specifically to chemical stimuli [74]; however, other studies with the 6-OHDA lesion mice model of ADHD showed increased sensitivity to both mechanical and thermal stimuli [75]. Neurogranin knockout mice (model of several human disorders) also showed hyperactivity and elevated pain sensitivity [76].

Despite the models’ differences, there was a consensus of a higher pain sensitivity associated with ADHD. In our hands, under the same conditions, dopaminergic lesions had a stronger effect on females. Interestingly, in our study, the control females showed a higher threshold for pain than males. This effect may not be DA-dependent because there are no sex-dependent differences in TH staining in the VTA [77,78]. However, although sex/gender differences in pain perception have long been observed in humans (reviews [79,80]), caution must be taken with rodent models because sex differences to different noxious stimuli vary greatly in different strains of rats and/or mice [81]. 

In females, one month of ABA treatment prevented the pain sensitivity induced by the 6-OHDA lesion. On the other hand, in males, ABA treatment had an overall effect in increasing thermal pain threshold, but it only increased the threshold of mechanical pain in sham mice. Altogether, these findings suggest a potential beneficial effect of ABA in alleviating pain sensitivity. This has been further supported by recent reports showing that ABA ameliorates neuropathic pain by reducing spinal cord inflammation [82]. Further studies are required to further evaluate the duration of intervention in males.

To evaluate whether the dopaminergic lesion affected brain inflammatory status underlying pain sensitivity, we carried out the morphological characterization of microglia in the ACC and posterior insular cortex (pIC) because the ACC-pIC connection is an important pain inhibitory circuit [83,84]. Moreover, ACC is considered a key region associating sex differences in the pain threshold [78]. Microglia morphology reflects its activity status, and more ramified microglia correspond to the resting/surveillance state, which is a characteristic of the healthy brain, whereas a less branched morphology corresponds to an activated (M1/M2) microglia status [85,86,87]. Further differentiation between M1 and M2 is beyond the present study. 

Our results indicated that, in two-month-old females and males, dopaminergic reduction by perinatal 6-OHDA lesion in the VTA induces proinflammatory microglia in one of its target areas, ACC. This result could be explained in line with the evidence suggesting that low dopamine levels can trigger inflammation by selectively stimulating high-affinity dopamine receptors [55]. Other studies have also demonstrated an association between inflammation in ACC and pain sensitivity [88]. 

Perinatal 6-OHDA injection increased activated microglia also in the pIC (target of ACC), but not in males. Because males did not show alterations in thermal or mechanical stimulus sensitivity, this result may suggest that inflammation in pIC correlates better with pain hypersensitivity. This finding is in line with studies using a rat model of nerve injury reporting that pharmacological inhibition of glia in pIC alleviated chronic pain [89].

We confirmed that a one-month treatment with ABA has an anti-inflammatory effect, but only in females, suggesting an important sex-dependent response to ABA. It remains to be determined whether longer treatments would also improve microglia inflammatory status in males, as we have observed in prior studies [46].

Oxidative stress and neuroinflammation are also closely related to ADHD [90]. Apurinic/apyrimidinic endonuclease/redox effector-1 (APE1) is a vital mediator in redox signaling, in addition to its DNA repair function [91]. Importantly, APE1 is considered a hub for inflammatory-oxidative connections [92]; thus, targeting it has been proposed as a therapeutic option, either by reducing [93] or by restoring it [94,95]. This paradoxical effect of APE1 may be because of its pleiotropic effects, the toxic stimulus, or the cell type. It could also be related to the window of time, given the fact that APE1 is engaged when oxidative stress occurs; however, the long-term reduced levels may increase vulnerability to stress. For example, in animal models of senescence, chronic low APE1 levels correlate with increased mitochondrial DNA damage [96].

We found that perinatal 6-OHDA lesions reduced APE1 expression in two-month-old females’ ACC and that this reduction was rescued by one month of ABA administration. This suggests that, in females, increased pain sensitivity is associated with lower APE1 levels together with higher inflammation in ACC. Additionally, other models of inflammatory pain have shown that APE1 levels are reduced in the spinal cord [50]. In males, the lesion interacted with ABA, reducing the APE1 levels. We did not observe a correspondence with behavior. This suggests a dissociation in males of pain sensitivity, neuroinflammation, and ACC. Further experiments are required to understand the sex-dependent interaction of neuroinflammation and oxidative stress

APE1 is upregulated by the brain-derived nerve factor (BDNF) [97], and we have demonstrated that ABA administration increases brain BDNF expression [57], suggesting a potential mechanism by which ABA could exerts an antioxidant action. Other studies using a rat model of aluminum-induced neuroinflammation showed that resveratrol, an antioxidant and anti-inflammatory molecule, increased APE1 levels [98]. Further studies are required to confirm whether longer ABA treatment can rescue APE1 levels in lesioned mice and to understand the interaction between dopaminergic dysfunction and ABA action. 

Although in pIC, perinatal 6-OHDA injection reduced APE1 expression in females and males, one month of ABA treatment did not rescue this alteration. Because ABA treatment rescued pain sensitivity in females, this suggests that the oxidative stress in pIC may not be strongly associated with the pain threshold. 

## 5. Conclusions

Our findings have supported the sex-dependent effect of perinatal 6-OHDA lesions, inducing higher pain sensitivity in females and increased spontaneous locomotor activity in males. Thus, our data may reflect some human characteristics, pointing to a higher risk of pain sensitivity in females and a higher risk of hyperactivity in males.

In females, pain sensitivity was associated with inflammatory microglia and oxidative stress in ACC and posterior IC (target of ACC). In males, inflammation was only seen in ACC, not in pIC, suggesting a sex-dependent effect of DA depletion. 

Importantly, in females, ABA treatment alleviated pain hypersensitivity (together with a reduction in inflammation and oxidative stress in specific brain areas). In males, ABA reduced hyperactivity (but did not affect neuroinflammation). 

Our data have strengthened the relationship of inflammation and oxidative stress in ACC-pIC underlying pain sensitivity in females and the potential beneficial role of ABA as a therapeutic intervention in ADHD management. Further studies are warranted to understand the sex differences in the responses to dopaminergic deficits and treatments. 

## Supplementary Materials

The following supporting information can be downloaded at https://www.mdpi.com/article/10.3390/cells12030465/s1, Figure S1: Percentage of TH expression Figure S2: Percentage of time spent in the open arms in the first 5 min.
