# Supplementary material for: Targeting Neuroinflammation with Abscisic Acid Reduces Pain Sensitivity in Females and Hyperactivity in Males of an ADHD Mice Model
Source: Cells. 2023 Jan 31;12(3):465. doi: 10.3390/cells12030465 (PMC9914171; doi:10.3390/cells12030465)
Supplement: Supplementary file 1 [file cells-12-00465-s001.zip › cells-2170251-supplementary.pdf]

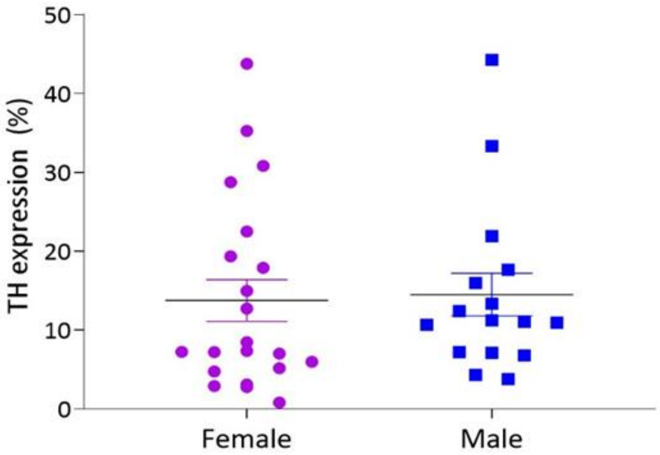

Supplementary Figure S1. Percentage of TH expression within the 6-OHDA injected group, at two months of age. In the study subjects with higher that 45% of TH expression (5 females and 6 males) have been removed.

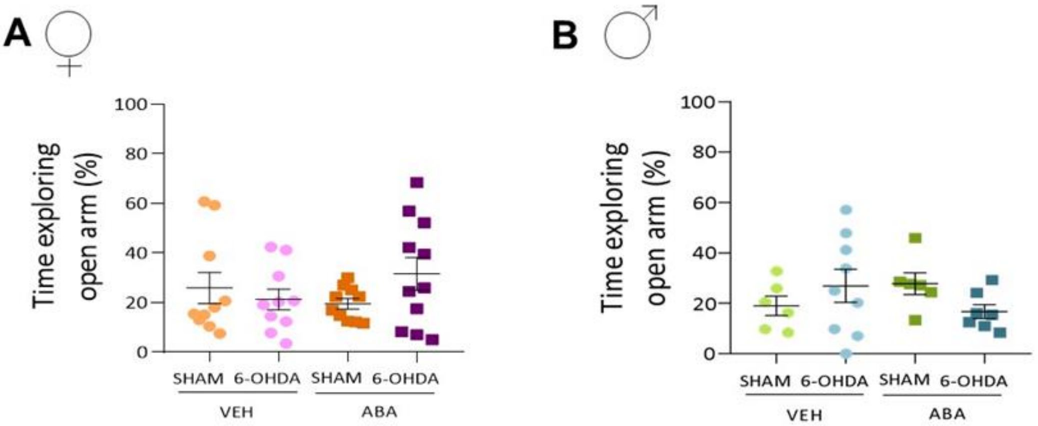

Supplementary Figure S2. Percentage of time spent in the open arms in the first 5 minutes of total time exploring. A) females. B) Males. Data is represented as the means ± SEM. And analyzed by two -Way ANOVA.
